# Estimating genome-wide DNA methylation heterogeneity with methylation patterns

**DOI:** 10.1186/s13072-023-00521-7

**Published:** 2023-11-09

**Authors:** Pei-Yu Lin, Ya-Ting Chang, Yu-Chun Huang, Pao-Yang Chen

**Affiliations:** 1 Institute of Plant and Microbial Biology, Academia Sinica, Taipei, 115 Taiwan; 2 Bioinformatics Program, Taiwan International Graduate Program, National Taiwan University, Taipei, 115 Taiwan; 3 Bioinformatics Program, Institute of Statistical Science, Taiwan International Graduate Program, Academia Sinica, Taipei, 115 Taiwan

**Keywords:** DNA Methylation pattern, Methylation heterogeneity, DNA methylation, Mathematical modelling, Bisulfite sequencing, Enzymatic methyl sequencing, Next Generation Sequencing, Epigenetics

## Abstract

**Background:**

In a heterogeneous population of cells, individual cells can behave differently and respond variably to the environment. This cellular diversity can be assessed by measuring DNA methylation patterns. The loci with variable methylation patterns are informative of cellular heterogeneity and may serve as biomarkers of diseases and developmental progression. Cell-to-cell methylation heterogeneity can be evaluated through single-cell methylomes or computational techniques for pooled cells. However, the feasibility and performance of these approaches to precisely estimate methylation heterogeneity require further assessment.

**Results:**

Here, we proposed model-based methods adopted from a mathematical framework originally from biodiversity, to estimate genome-wide DNA methylation heterogeneity. We evaluated the performance of our models and the existing methods with feature comparison, and tested on both synthetic datasets and real data. Overall, our methods have demonstrated advantages over others because of their better correlation with the actual heterogeneity. We also demonstrated that methylation heterogeneity offers an additional layer of biological information distinct from the conventional methylation level. In the case studies, we showed that distinct profiles of methylation heterogeneity in CG and non-CG methylation can predict the regulatory roles between genomic elements in Arabidopsis. This opens up a new direction for plant epigenomics. Finally, we demonstrated that our score might be able to identify loci in human cancer samples as putative biomarkers for early cancer detection.

**Conclusions:**

We adopted the mathematical framework from biodiversity into three model-based methods for analyzing genome-wide DNA methylation heterogeneity to monitor cellular heterogeneity. Our methods, namely MeH, have been implemented, evaluated with existing methods, and are open to the research community.

**Supplementary Information:**

The online version contains supplementary material available at 10.1186/s13072-023-00521-7.

## Background

### Measuring cellular heterogeneity with DNA methylation heterogeneity

**Fig. 1:**
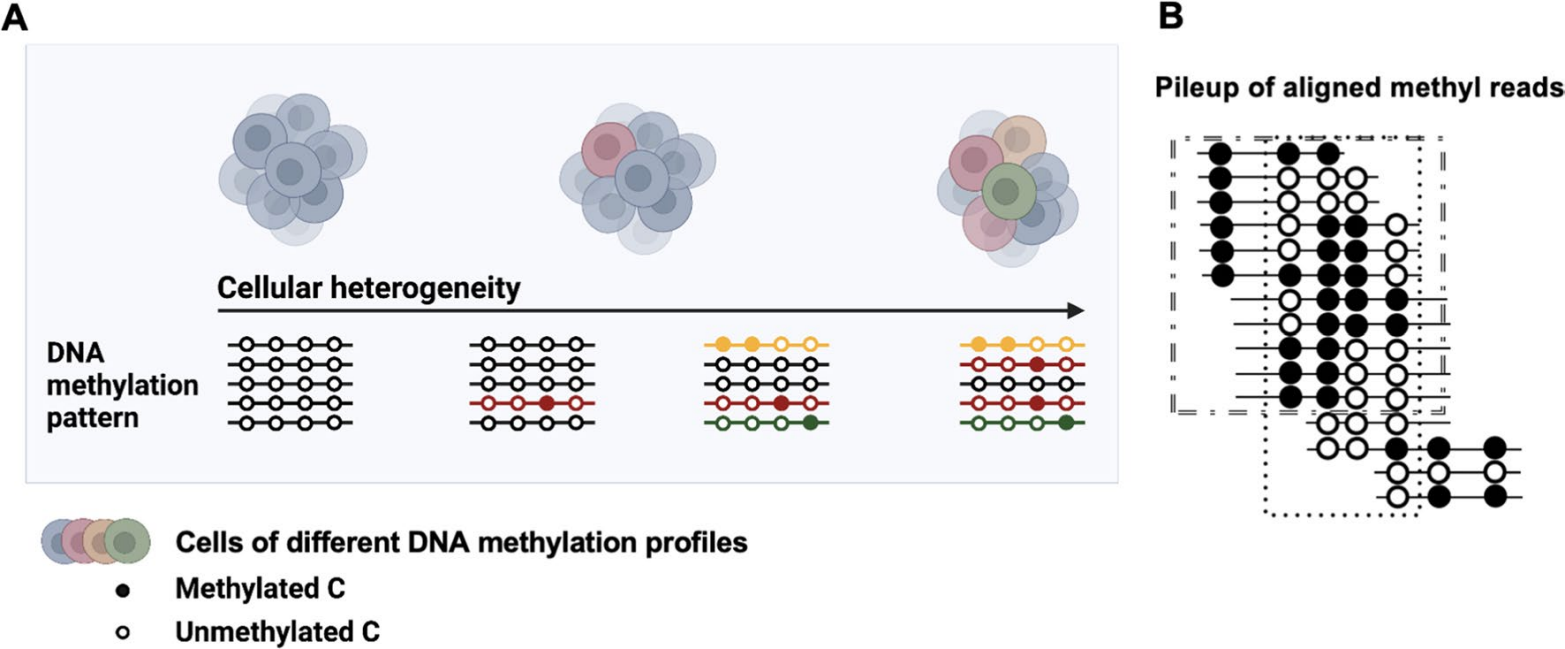
Illustrations of the DNA methylation patterns as a proxy for monitoring cellular development. **A** DNA methylation patterns are used to monitor cellular heterogeneity, possibly caused by cellular development, cell-type mixtures, differentiation, genetic changes, diseases, stresses. The black (white) dots represent methylated (unmethylated) cytosines. The different colours correspond to different subtypes of cells (Created with BioRender.com). **B** DNA methylation patterns formed by aligned methyl reads (by BS-seq or EM-seq) with colour-coded methylation statuses. A set of methylation haplotypes are circled to indicate the reads considered by certain scores covering a specific locus

DNA methylation as a heritable epigenetic modification that occurs at cytosines, plays critical roles in many biological processes, such as transcriptional regulation, developmental programming [1], and disease progression [2]. Genome-wide DNA methylation and its associations with gene expression have been extensively studied with the most recent next-generation sequencing [3] approaches, such as bisulfite sequencing (BS-seq) [4, 5] and enzymatic methyl sequencing (EM-seq) [6]. The methylation status (methylated or unmethylated) at a specific cytosine can be established when reads converted from methyl reads data (*i.e.*, reads from BS-seq or EM-seq) are aligned to the reference genome. In bulk methylation sequencing such as BS-seq and EM-seq, millions of cells are pooled, representing mixtures of cells that are likely heterogeneous that can be linked to their variable DNA methylation profiles (Fig. 1A). Aligned methyl reads within a given genomic region yield methylation patterns formed by rows of multiple cytosines, representative of individual cells (Fig. 1B). The methylation patterns at genomic regions may range from completely methylated to completely unmethylated. The intermediate patterns could indicate variations in DNA methylation among the cells.

DNA methylation heterogeneity at a specific locus is defined as the variation amongst DNA methylation patterns at this locus, within a pool of cells. Methylation heterogeneity may result from a variety of epigenetic regulations from genetic or epigenetic factors [7]. For example, the variable methylation at promoters is often associated with the transcriptional responses to environmental stimuli or cellular development progression [8, 9] (Fig. 1A). As the behaviour of individual cells within a population may not be identical, this may be due to genetic changes that are often accompanied by epigenomic changes or, in the case of cellular differentiation, epigenetic changes. By monitoring the variation methylation patterns, methylation heterogeneity might be able to capture the fingerprints of the genetic or epigenetic factors during the biological development or disease progression.

### Experimental approaches and computational methods for assessing heterogeneity

Both experimental approaches and computational methods have been developed for assessing methylation heterogeneity. Among the experimental strategies, single-cell BS-seq (scBS-seq) was developed to mainly study the methylation heterogeneity of rare cells (*e.g*., oocytes) of mice [7, 10]. scBS-seq enables direct measurement of methylation heterogeneity, through single-cell bisulfite sequencing followed by computational analysis using tools such as BPRMeth [11], Melissa [12], and scMET [13], to impute and cluster single-cells by their methylation profiles. Still, scBS-seq comes with its own costs and challenges, such as the requirement to isolate individual cells in the preparation of libraries, low read mapping ratios, and high costs due to the number of cells that must be sequenced [7], and significant loss of DNA due to bisulfite treatment. Moreover, the original protocols for sequencing single mammalian cells are not directly applicable to sequencing single plant cells due to the larger cell size of plant cells and their cell wall, hampering plant research. Although DNA methylation profiling using third-generation sequencing techniques does not require bisulfite conversion [14, 15], the high sequencing error rate has been reported to be over 15% for base calling [15] and up to 40% for methylation calling [16]. Therefore, attempts have been made to quantify methylation heterogeneity computationally using the methylation statuses of cytosines at genomic regions from pooled cells of methyl-seqs.

Up to date, only a few computational methods [3, 17–23] and databases [24] have been developed for estimating methylation heterogeneity using genome-wide methylation data from pooled cells (see Table 1 for a list of methods and their features). Most of them were devised to compare the number of methylated and unmethylated cytosines or read pairs that were concordantly or discordantly methylated within a genomic region (Methylation-concurrence; MC [22], Proportion of Discordant Reads; PDR [20], Fraction of Discordant Read Pairs; FDRP [18]), with extensions to probability-based setting (Epipolymorphism; EP [19]) or to incorporate pattern similarity (Quantitative FDRP; qFDRP [18], Methylation Haplotype Load [21]; MHL). MHL considers pattern similarity as it calculates the fraction of substrings of all possible lengths that are fully methylated in each of the reads, which also makes it share similar characteristics to DNA methylation level [18]. qFDRP compares the similarity of read-pairs, and weighs higher for discordant pairs potentially from intermediately methylated regions. Hence the qFDRP score may not be completely independent of methylation levels. In addition, Shannon entropy-based approach was devised to estimate the degree of chaos analogous to the heterogeneity (Methylation entropy; ME [23]).

**Table 1:** Computational methods for scoring genome wide DNA methylation heterogeneity

Method	Formula		Approach	Applicable to non-CG sites	Consideration of pattern similarity	Linearity of the score	Independent of methylation level^1^	Genome-wide screening
Model-based (MeH)								
Abundance based	${\left(\sum_{i=1}^{R}{a}_{i}^{2}\right)}^{-1}\in \left\{1,\dots ,{2}^{w}\right\}$	$a:$ methylation patterns	Counting distinct methylation patterns	●		●	●	●
Pairwise-similarity based	${\left(\sum_{i=1}^{R}\sum_{j=1}^{R}{{d}_{ij}p}_{ij}^{2}\right)}^{-1/2}>0$	$p:$ methylation patterns	Considering pairwise similarity between patterns	●	●	●	●	●
Phylogenetic-tree based	${\left(\sum_{i=1}^{B}{L}_{i}{a}_{i}^{2}\right)}^{-1}>0$.5	$a:$ methylation patterns	Considering the total similarity among all patterns	●	●	●	●	●
Other methods								
Methylation-concurrence [18, 22]	$\frac{\sum_{c=1}^{C}{\omega }_{c}}{\sum_{c=1}^{C}{\omega }_{c}+ \sum_{m=1}^{M}{\omega }_{m}+ \sum_{u=1}^{U}{\omega }_{u}}\in [\mathrm{0,1})$	$\omega :$ reads covering CG sites	Measuring the methylation concurrence between patterns				●	
Proportion of Discordant Reads (PDR) [18, 20]	$\frac{{\sum }_{r\in {R}_{c}}I(\exists i,j \epsilon r \mathrm{s}.\mathrm{t}. {x}_{j,r}\ne {x}_{i,r})}{|{R}_{c}|}\in [\mathrm{0,1}]$	$r:$ reads covering CG sites	Counting distinct methylation patterns among reads				●	●
Methylation entropy [17, 18, 23, 24]	$\frac{1}{w}\sum_{k}-{a}_{k}{log}_{2}{a}_{k}\in [\mathrm{0,1}]$	$a:$ methylation patterns	Measuring the chaos among the reads of different methylation patterns				●	●
Epipolymorphism [18, 19]	$1-\sum_{k}{a}_{k}^{2}\in [\mathrm{0,1})$	$a:$ methylation patterns	Estimating the probability of observing two different patterns at random				●	●
Fraction of Discordant Read Pairs (FDRP) [18]	$\frac{{\sum }_{{r}_{s}\in {R}_{c}}{\sum }_{{r}_{t}\in {R}_{c},t>s}I(\exists i\in \left\{{r}_{s}\cap {r}_{t}\right\} \mathrm{s}.\mathrm{t}. {x}_{i,{r}_{s}}\ne {x}_{i,{r}_{t}})}{(\genfrac{}{}{0pt}{}{|{R}_{c}|}{2})}\in [\mathrm{0,1}]$	$r:$ reads covering CG sites	Calculating pairwise disagreement of between any two reads				●	●
Quantitative FDRP (qFDRP)[18]	$\frac{{\sum }_{{r}_{s}\in {R}_{c}}{\sum }_{{r}_{t}\in {R}_{c},t>s}\frac{{\sum }_{i\in {\{r}_{s}\cap {r}_{t}\}}I({x}_{i,{r}_{s}}\ne {x}_{i,{r}_{t}})}{|\{{r}_{s}\cap {r}_{t}\}|}}{(\genfrac{}{}{0pt}{}{|{R}_{c}|}{2})}\in [\mathrm{0,1}]$	$r:$ reads covering CG sites	Quantifying the similarity of paired-methyl reads by Hamming distance		●			●
Methylation Haplotype Load (MHL) [18, 21]	$\frac{{\sum }_{l=0}^{L}(l+1)\frac{{\sum }_{r\in {R}_{c}}{\sum }_{i=1}^{\left|r\right|-l}I({x}_{i,r}=1\wedge \dots \wedge {x}_{i+l.r}=1)}{{\sum }_{r\in {R}_{c}}\left|r\right|-l}}{{\sum }_{l=0}^{L}l+1}\in [\mathrm{0,1}]$	$r:$ reads covering CG sites	Estimating the fraction of strings that are fully methylated for all possible lengths		●			●

^1^See method description above (by the formula, and the designing principle and the literature)

While these methods share differences in their own ideas and strength, the evaluation of methods is associated with the nature of DNA methylation and the data format of next-generation sequencing. Therefore, here is a list of suggested features to be considered in their implementations. Firstly, the capability of analysing methylation at non-CG sites, *i.e.*, cytosines of CHG and CHH contexts (H = A, C, or T). In fact, non-CG sites outnumber CG sites on the genomes of both plants and animals. In plants and even fungi, non-CG methylation has been known to play critical roles in many important biological processes [1]. Secondly, the scoring linearity, which represents a linear correlation between the score and the underlying methylation heterogeneity. Linear scoring enables a fair assessment of heterogeneity across all genomic regions, and loci of different heterogeneity between samples. As shown in Additional file 1: Fig. S1, non-linear scoring is less likely to faithfully reflect the underlying heterogeneity of loci between low and high heterogeneity (*i.e.*, skewed). Thirdly, the consideration of similarity between different methylation patterns. The cells sharing the same methylation patterns are likely from the same cell subpopulation. Two highly similar patterns may result from a gradual change of methylation initiated from a few cytosines of cells within the same population. Disregarding the pattern similarity by treating all patterns distinct may lose the subtle information on cellular development [25]. Fourthly, confounding methylation heterogeneity with the methylation level in the scoring; such scoring can be easily confused with the estimates of methylation level in which some patterns are given more weight than others (*e.g.*, fully methylated vs. unmethylated). The scoring that confounded methylation level can be diverted away from the original idea of estimating methylation heterogeneity in which all patterns are equally considered. Lastly, the capability of genome-wide screening is particularly important for the user community. The implementation of the methods should allow genome-wide screening and the comparison between multiple samples, so to enable the detection of loci with variable heterogeneity. Following these feature considerations for methylation heterogeneity estimators, we have summarized a feature table covering several popular implementations (see Table 1).

### Estimation of methylation heterogeneity based on a biodiversity framework

In this study, we introduce a family of diversity indices based on a mathematical model by Chao et al*.* [26] that has proven to be successful in quantifying biodiversity. Biodiversity can be interpreted as the effective number of species or types. We adopted this framework and its specific variant models to quantify methylation heterogeneity. In Chao et al*.*’s [27] model (see Eq. 1), Hill numbers [28], or the effective number of types, are a parametric family of diversity indices of order *q*, which refer to the number of equally abundant types. The Hill number is needed for the average proportional abundance of the types to be equal to that observed in the dataset of interest. In Chao et al*.*’s framework, set $C$ was considered as a collection of entities. For each entity $u$ in $C$, its attribute value was given by ${v}_{u}$, and its abundance was given by ${a}_{u}$. The total abundance of entities in $C$ was given by the sum of attributes weighted by their corresponding abundances, $\overline{V }= {\sum }_{u\in C}{v}_{u}{a}_{u}$. Therefore, the relative abundance of entity $u$ is $\frac{{a}_{u}}{\overline{V} }$, and the sum of the products of attributes and their corresponding abundance of all entities in $C$, equals to 1, ${\sum }_{u\in C}{v}_{u}\left(\frac{{a}_{u}}{\overline{V} }\right)=1$. The attribute diversity of set $C$ (with order $q$) based on a specific attribute was given by a unified framework as follows:

1 $${}^{q}AD\left(\overline{V }\right)={ \left[\sum_{u\in C}{v}_{u}{\left(\frac{{a}_{u}}{\overline{V} }\right)}^{q}\right]}^{\frac{1}{1-q}} .$$

where $q\ne 1$ is the parameter that determines the sensitivity of the model to relative abundances. When$q=0$, the abundances of the attributes do not contribute to the formula, and $q=1$ gives the exponential of Shannon entropy [29] when ${v}_{u}=1$ for all entities$u$, which weighs attributes according to their abundances. When$q=2$, it is the reciprocal form of the Simpson index [30], which is found to provide a robust estimate of diversity in different situations. By varying set $C$ and attribute value${v}_{u}$, Chao et al*.* presented a unifying framework to cover major variants of Hill numbers, based on different attributes for the quantification of diversity, including species diversity [31], phylogenetic diversity [28] and the distance-based functional diversity [27].

When this base model (Eq. 1) is used in measuring biodiversity, set $C$ is considered as a collection of different species $u$, and the attribute value of this species ${v}_{u}$ is a function of the species $u$ to describe for example the population size of the species, or the relative similarity to another species, or to all species in the collection. The abundance of the species ${a}_{u}$ would be just the population size of the species. The other variables as described above are either normalising factors, or the model parameters not directly associating with the species.

Likewise, when considering methylation patterns observed at a specific locus, such as a genomic region, we hypothesized that estimating methylation heterogeneity at this locus is analogous to measuring biodiversity within a specific field. In a simple setting without considering the similarity between methylation patterns, the variables in the base model (Eq. 1) in methylation heterogeneity can then be translated as:

1. Set $C$ is considered as a collection of different methylation pattern $u$ observed from the alignment at a specific locus; and
2. ${v}_{u}$ is the attribute value of pattern$u$. It can be the abundance of the pattern $u$, or in the extended setting, the pairwise similarity between patterns, or amongst all observed patterns in the alignment (see Methods; Eqs. 3–6);
3. ${a}_{u}$ is the abundance of the pattern $u ,$ and could be estimated as the number of reads having this pattern$u$.

Such setting can be modified to accommodate the pattern similarity, using pattern pair instead of pattern as the entity $u$ as well as the corresponding attribute value.

Chao et al*.’*s mathematical base model (Additional file 1: Note S1) possessed several mathematical properties, such as scale invariant, weak monotonicity and doubling property. Among them, the weak monotonicity refers to that the diversity should increase when adding a new species largely diversified from the current ones [32]. The doubling property refers to the characteristic that mixing two mutually exclusive groups of the same diversity in equal weights results in the doubling of diversity or the quadrupling of distance-based diversity [27]. The doubling property is considered a fundamental characteristic required for diversity estimation. Please note that while all variants based on the Hill number exhibit the doubling property, neither Shannon entropy [29] nor the Simpson index [30] shows this property.

We found that the mathematical properties in Chao et al*.*’s framework of Hill numbers (Additional file 1: Note S1) are ideal for quantifying methylation heterogeneity using methylation patterns for several reasons. First, measuring epigenetic diversity with methylation patterns is analogous to measuring the biodiversity of species, and the mathematical models are conceptually novel in analysing methylation heterogeneity. Second, the mathematical properties and their meanings make the heterogeneity scoring more interpretable and comparable among samples; therefore, we adopted these properties to estimate DNA methylation heterogeneity. As a result, we demonstrated that our three model-based methods of methylation heterogeneity-abundance based (AB), pairwise-similarity based (PWS), and phylogenetic-tree based (PHY) can help overcome the shortcomings of existing methods by analysing synthetic data, and benchmarked by scBS-seq data. Lastly, we provided examples showing the strength of this approach for profiling non-CG methylation heterogeneity in *Arabidopsis*, and monitoring disease progression in cancer samples. Moreover, the computational programs of our models are written to implement the genome-screening estimation of methylation heterogeneity (referred to as MeH [33] and compare samples obtained under different conditions. Our implementation of MeH and the tutorial are publicly available at https://github.com/PaoyangLab/MeH.

## Results

We first demonstrate the behaviour of both the existing methods and our proposed methods with toy examples of synthetic alignments. Subsequently, to demonstrate a major characteristic of scoring, *i.e.*, the linearity, we merge multiple single-cell methylomes to test if the estimates of heterogeneity increase with the number of cells. In addition, we showed that comparing methylation heterogeneity can reveal differences between samples that might not be detectable by looking just at methylation levels. Finally, to test our methods on real data, we analyzed *Arabidopsis* methylome to profile non-CG methylation heterogeneity and human colorectal cancer data.

### The behaviour of different scores in evaluating heterogeneity

We compiled a table of evaluation for several popular existing methods and our model-based methods (Table 1). The table includes specific features to be considered in methylation heterogeneity estimation and implementation. Overall, the main advantages of our model-based methods over existing methods lie in the possible extension to non-CG methylation sites, scoring linearity and the consideration of similarity between methylation patterns for unbiased and meaningful evaluation.

**Fig. 2:**
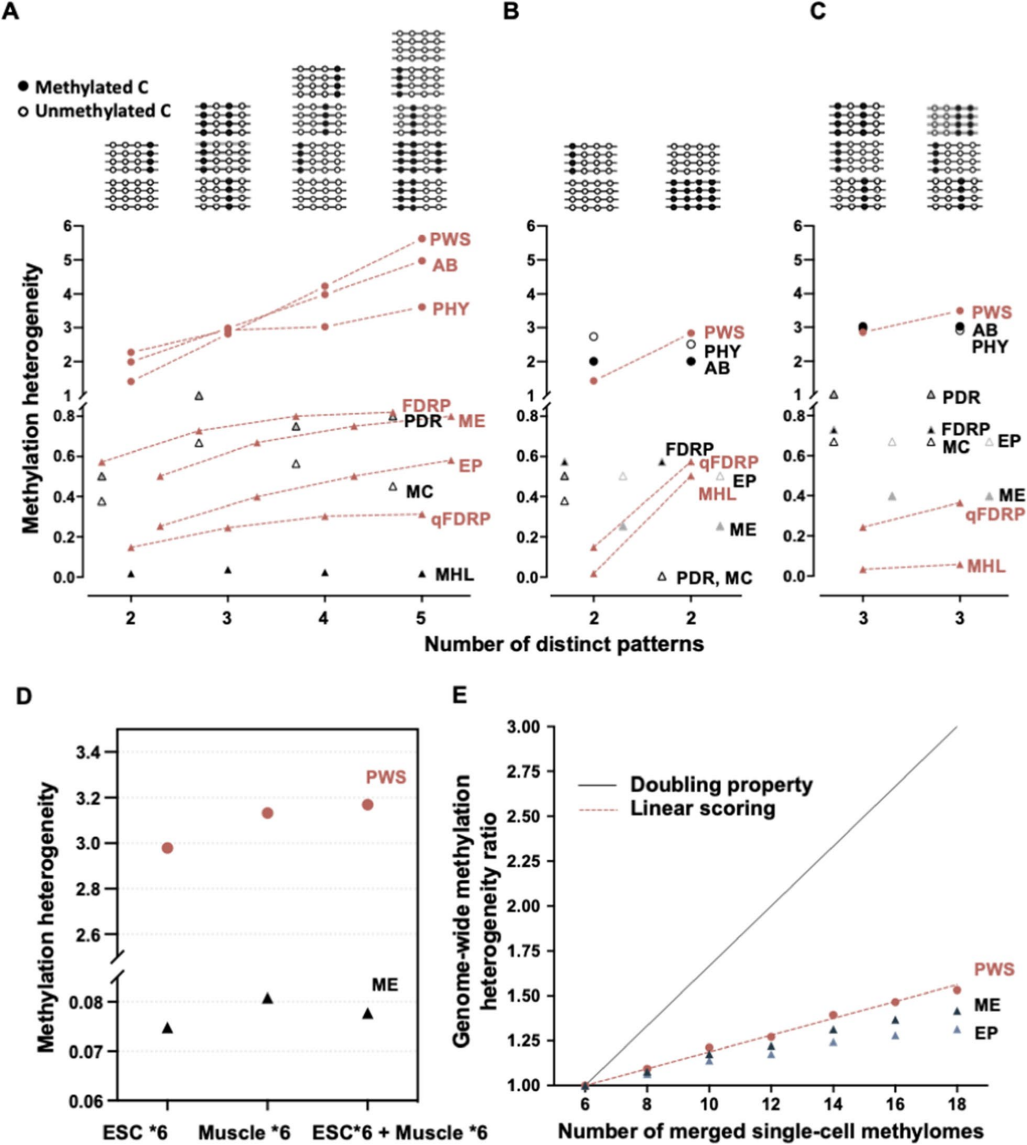
Evaluation of methylation heterogeneity methods. **A**–**C** Estimating methylation heterogeneity with synthetic datasets. Top panel lists combinations of methylation patterns at different loci. Circles are model-based methods, and triangles are existing methods. Dashed lines represent the methods with increasing trends. Four types of scores are used in the comparisons, model-based methods: AB, PWS and PHY; accordance-based methods: MC, PDR, FDRP; Entropy-based: ME, and probability-based: EP; Existing methods considering pattern similarity: qFDRP and MHL. **D** The methylation heterogeneity of merged mouse ESC and muscle single-cell methylome estimating by PWS and ME. **E** Genome-wide methylation heterogeneity ratios are plotted against different numbers of ESC single-cell methylomes. The black line represents the expected values given merged cells are all heterogeneous while the red represents linearity

To assess the ability of our proposed method and other existing methods to precisely detect changes in methylation heterogeneity, we created toy examples with variable methylation patterns. To ensure a fair comparison between the methods, fully aligned reads are constructed to represent complete methylation patterns, and combinations of methylation patterns resembling alignments at a genomic region are simulated (see Fig. 2A–C, top panels). Firstly, we tested the hypothesis that the methylation heterogeneity would increase as new patterns occur. As a result, our three proposed models, as well as FDRP, qFDRP, MHL, ME, and EP showed such monotonic increasing trends (see dashed lines in Fig. 2A), while other methods showed differently. Next, we examined the importance of pattern similarity in the models, given that methylation patterns likely result from gradual changes associated with methylation maintenance. As shown in Fig. 2B and C, we would expect the ideal scores to increase when the patterns became more diverse (from left hand to right hand). We found that only PWS, MHL, and qFDRP were able to detect such changes in methylation patterns (see Fig. 2B and C). As a result, only two methods, PWS and qFDRP, aligned with both hypotheses. One specific concern for qFDRP is that the design of its score makes it easily influenced by the methylation level as described earlier (see Table 1). Our model-based method, specifically the PWS approach, demonstrated the ability to balance all these features effectively. Therefore, PWS was used for the following analyses.

### Evaluating the scoring linearity using single-cell methylomes

When examining the real data, we expected the level of methylation heterogeneity to increase as new patterns are introduced. We processed a number of single-cell methylomes of mouse [10] from two different cell types, muscle cells and embryonic stem cells (ESC). We used the PWS method for the analysis as it is the only method that passed the previous evaluations in Figs. 2A–C. We also included ME and EP in the single-cell analysis as they share a similar data input format for such genome-wide analysis.

We would expect the methylation heterogeneity scores to increase from a pooled single-cell methylomes of one cell type to those of mixed two cell types. To this end, six single-cell methylomes of two cell types were pooled before the heterogeneity was estimated (*i.e.*, the raw reads from multiple single-cell methylomes were pooled to form one merged methylome before they were aligned to the reference genome). We found that as expected methylation heterogeneity from PWS is increased in the mixed cell types (see Fig. 2D). In contrast, running the ME method on the same dataset, the mixed cell types showed a lower methylation heterogeneity score.

Next, we evaluated the methylation heterogeneity between different numbers of merged ESC single-cell methylomes for which we knew the compositions (see Fig. 2E). In a perfect setting, adding more cells of the same type would not increase heterogeneity. However, in this case of real data from ESC, each single cell methylome may not cover all expected patterns of ESC; based on the largely damaged DNA due to bisulfite treatments, the observed patterns may be very different between these single cell methylomes. Therefore, the methylation heterogeneity scores are likely to increase as the new patterns (from newly added single cells even from the same cell type) are added, we also expect to observe a gradual saturation of the methylation patterns, with the heterogeneity plateauing. First, different numbers of single-cell methylomes, *i.e.,* 6, 8, 10, 12, 14, 16, and 18, were combined as merged methylomes to mimic bulk sequencing data. On those methylomes merged from many cells we would expect overall a higher methylation heterogeneity (indicative of cellular heterogeneity) than those from fewer cells.

We computed the genome-wide methylation heterogeneity ratio for each of the selected methods (for details of the procedure and calculations Additional file 1: Note S3). Overall, we observed a monotonic increase in the ratios as the number of single-cell methylomes increased with all methods. The extrapolated line (red) from 6 to 8 methylomes was drawn for PWS to demonstrate the expected linear increases per every 2 methylomes added. This line also revealed that ME and EP are likely to reach their plateaus quickly that are clearly deviated from being linear, suggesting that the two scores were less sensitive in detecting new patterns. The lower sensitivity revealed by such nonlinearity in real data application is less favoured (see Additional file 1: Fig. S1 for demonstration), particularly when different samples or regions were compared. Additionally, we found that none of these methods perfectly displayed the doubling property (see Fig. 2E black solid line). This could have occurred because in real data these single cells of ESC are typically not mutually exclusive groups. Still, we found the PWS heterogeneity is relatively linear compared to other methods. It also showed less deviation when the number of single-cell methylomes increased, making it a plausible scoring.

### Comparing between methylation heterogeneity and methylation level

**Fig. 3:**
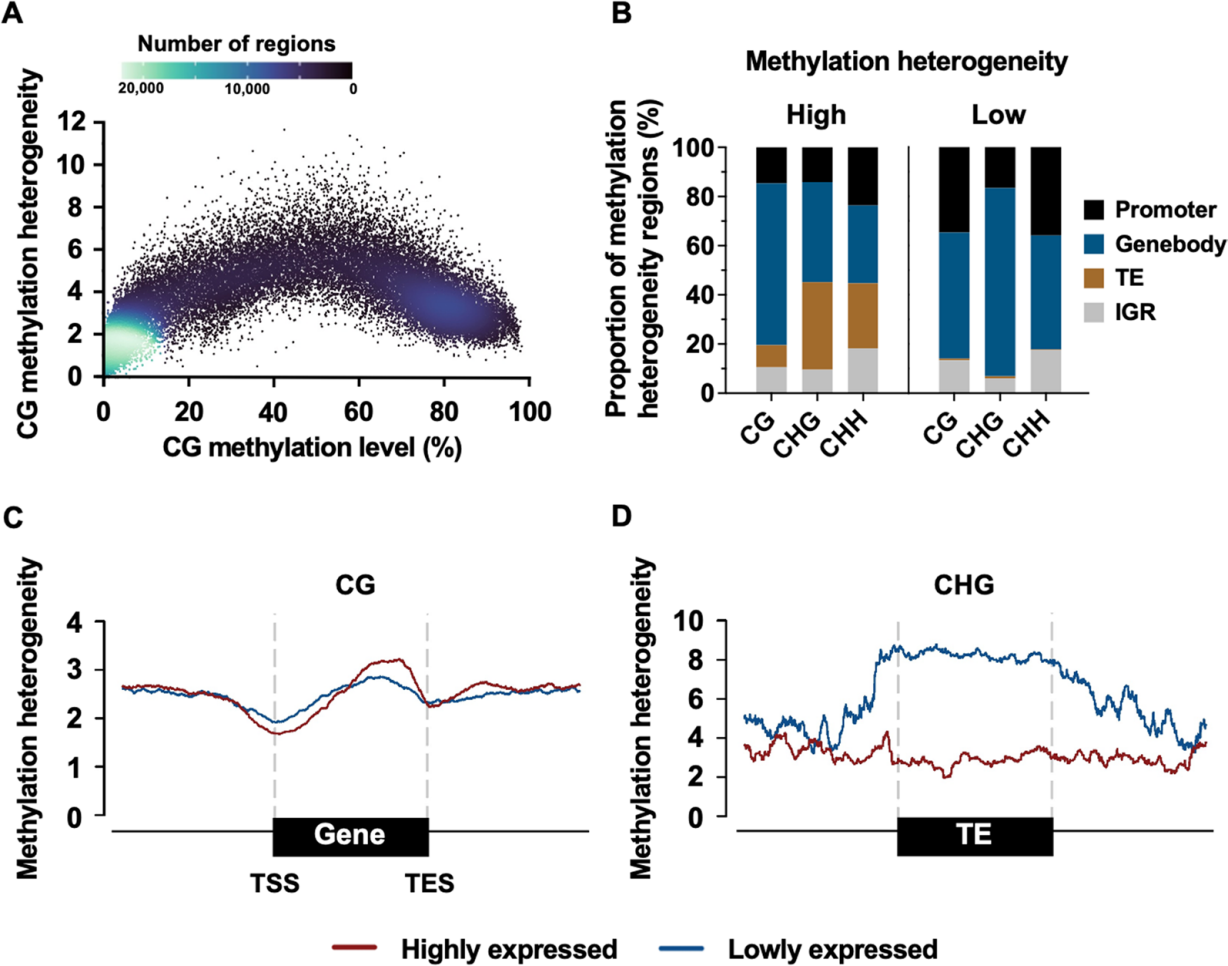
Genome-wide methylation heterogeneity profiles. **A** Mean methylation heterogeneity plotted with mean methylation levels in 3 replicates of adjacent normal samples of CRC. **B** Proportion of high (top 10%) and low (bottom 10%) methylation heterogeneity regions across different genomic features in *Arabidopsis thaliana* genome. **C** Metagene plot of *A. thaliana* CG methylation heterogeneity profile between highly and lowly expressed genes (top and bottom 25%). **D** Meta plots of *A. thaliana* CHG methylation heterogeneity between highly and lowly expressed TEs and their neighbouring regions

To determine the differences between methylation heterogeneity and the commonly used metric of methylation levels, we plotted the methylation heterogeneity estimated by the PWS method against the methylation levels of 3 replicates of samples from the human colorectal cancer (CRC) (Fig. 3A) and of *Arabidopsis* wild type methylome (Additional file 1: Fig. S2). As illustrated in Fig. 3A, the scatter plot indicated that the relationships between methylation heterogeneity and methylation varied across different cytosine contexts (*i.e.*, CG, CHG and CHH, H = A, C or T). We observed a curve-shaped relationship between methylation heterogeneity and methylation level at regions of CpG methylation, that the regions with higher methylation heterogeneity have intermediate methylation levels that are found in both in human and in *Arabidopsis*. These regions are likely to reflect a dynamic process of epigenomic changes that are commonly observed in genic regions (Fig. 3C). In *Arabidopsis* we also profiled the methylation heterogeneity at non-CG sites. We found that the non-CH sites (i.e., CHG and CHH) showed very different relationship with methylation levels comparing to the CG sites (Additional file 1: Fig S2). While non-CG sites are low methylation, a fraction of them are highly methylated alone with a higher methylation heterogeneity. It is important to note that moderately methylated regions across all contexts retain a diverse range of heterogeneity, which could be easily overlooked when performing the evaluation using the methylation level alone. Furthermore, our PWS scores were able to detect the changes in methylation heterogeneity when the changes in methylation levels were not apparent. In brief, methylation heterogeneity could potentially complement the use of methylation levels for identifying minor changes that cannot be detected using methylation levels, and provides a different layer of biological information from the conventional methylation level.

### Methylation heterogeneity in plant at CG and non-CG sites

To reveal the genome-wide methylation heterogeneity in plants, we employed PWS to analyze an *Arabidopsis* wild-type methylome with a coverage of 58X [34]. We found that the regions of high methylation heterogeneity preferentially targeted transposable elements (TEs) in both CG and non-CG sites, which is different from regions with low methylation heterogeneity (Fig. 3B). In addition, the high methylation heterogeneity at CG sites are largely enriched at genebody comparing to non-CG sites; suggesting a differential preference between CG and non-CG. Subsequently we compare the methylation heterogeneity at genes and TEs of high and low expression (top and bottom 25%), see Fig. 3C and D. We observed a negative association between CG methylation heterogeneity and gene expression near transcription start sites (TSS), followed by a positive association toward the transcription end sites (TES); which indicates the dynamic epigenetic regulation of DNA methylation at promoter and genebody. We also found that lowly expressed TEs exhibited higher CHG methylation heterogeneity compared to highly expressed TEs (see Fig. 3D), suggesting that the methylation patterns at active TEs are highly variable in plant cells.

Our study produced the first map of methylation heterogeneity in the plant. High methylation heterogeneity regions were identified to be located at specific genomic features, which differed between CG and non-CG methylation heterogeneity. Methylation heterogeneity was demonstrated to be linked with transcriptional regulation. Our results illuminated the unique functions of CG and non-CG methylation heterogeneity in the *Arabidopsis* genome.

### Strong association between genes with differential methylation heterogeneity and colorectal cancer-related diseases

Next, we wanted to demonstrate that the genomic regions with differential methylation heterogeneity may also be considered as biomarkers for phenotypes of interest. We downloaded and processed the human Reduced Representation Bisulfite Sequencing (RRBS) methylome data from CRC [35], which consisted of different stages, including stage III-IV CRC frozen tumours (tumour), normal-appearing mucosa as indicated by pathogens from the same patients (normal), and histologically confirmed matched normal samples collected from the margins on either side of the resected tumour (adjacent normal). The original study analyzed 10 samples per stage and found that the promoter methylation at specific cancer genes raised 40% to trigger the transcriptional changes at tumours, whereas at the adjacent normal the promoter methylation was only increased by 20% with no changes in expression, likely due to the lower changes in promoter methylation insufficient for triggering transcriptional changes.

As a demonstration of our method, we used 3 replicates from each normal, adjacent normal and tumour samples for methylation heterogeneity analyses using PWS method. The goal was to see if our PWS method is able to identifying putative biomarkers, as an alternative approach to the current approaches such as EWAS. A number of DNA methylation level studies have already shown that there existed methylation differences between say normal and normal-adjacent tissue [36] or between normal tissue and normal-tissue at risk of cancer development [37]. Therefore, we analyzed DNA methylation level in parallel, to assess the predictability between methylation heterogeneity and methylation level (Additional file 1: Fig. S4 for Venn diagram of differentially methylated regions, DMRs and differentially heterogeneous regions, DHRs).

**Fig. 4:**
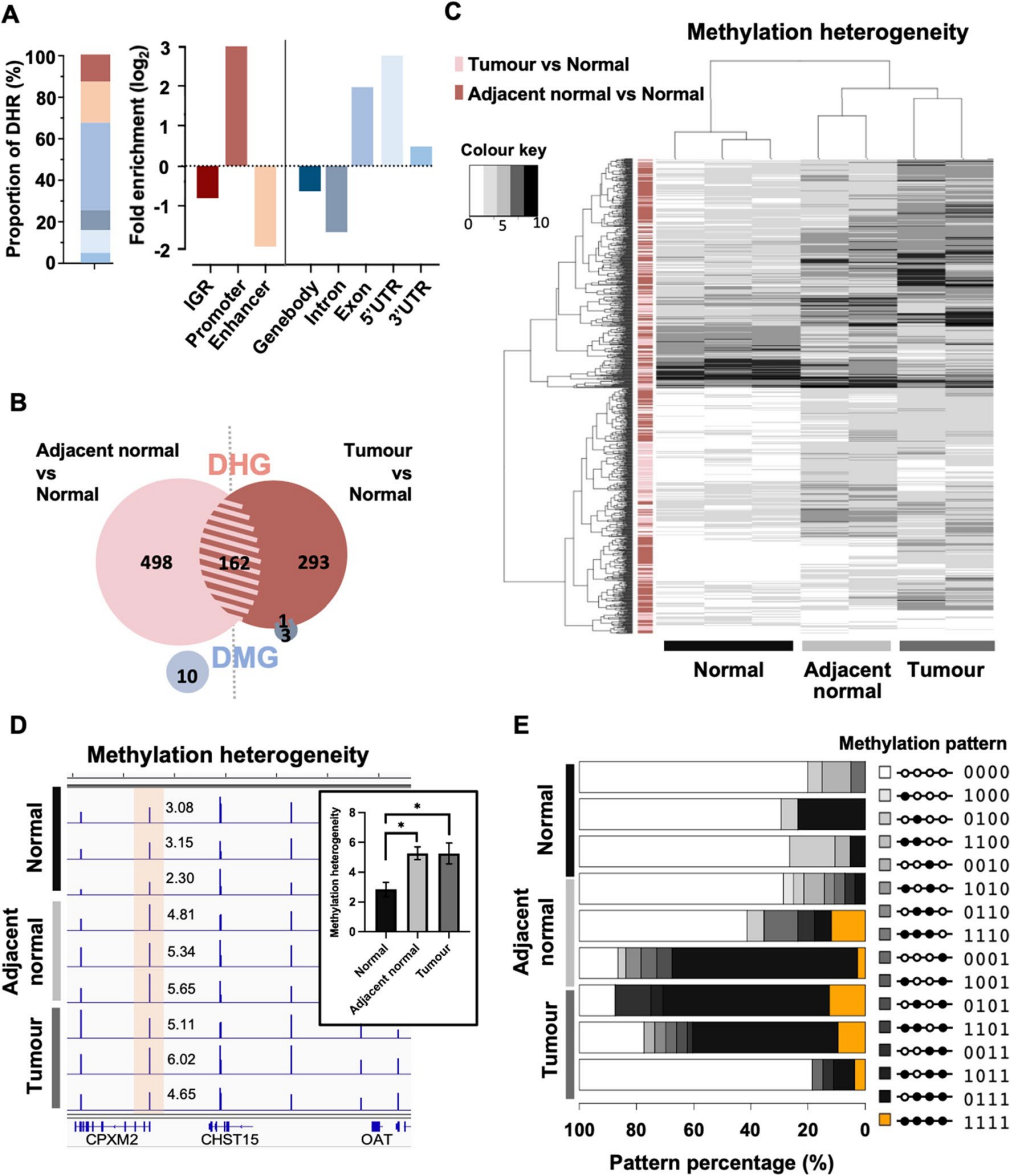
Comparison between methylation heterogeneity and methylation levels and the evaluation of PWS heterogeneity. **A** The proportion (left) and the enrichment plot (right) of DHRs at different genomic features. **B** Venn diagram of DHGs and DMGs. **C** Methylation heterogeneity heatmap of DHGs in the 3 stages of CRC samples. **D** IGV illustration of methylation heterogeneity estimated using PWS around CPXM2, with the DHR shaded in orange. Each blue bar indicates the mean methylation heterogeneity in bins of 400 bp, and the exact values of the bars at the DHR are labelled. **E** Composition of methylation patterns in all samples within a specific CG window 4 within the DHR from **D** for identifying a potential disease pattern

In total 2,319 DHRs are identified between adjacent normal and normal (n = 911), and between tumour and normal samples (n = 1,558). These DHR are mostly found at genebody (Fig. 4A, left-panel). After normalising against RRBS genome we found the DHR are enriched at promoters, exons, 5’- and 3’ UTR but not from introns; suggesting a possible association with transcription.

After we associated the DHRs with the genes that are co-localized with, 953 differentially heterogeneous genes (DHGs) are identified (Fig. 4B), whereas only 14 differentially methylated genes (DMGs) can be detected (15% methylation change and *p*-value < 0.05), including 2 genes C9orf69 and RAPGEFL reported in the original study (see Methods for identification of DHGs and DMGs). There is only one DMG, namely FK506-binding protein 10 (FKBP10), found to be also a DHG. This may suggest that the methylation level- and heterogeneity-based analyses actually targeted different sets of genes.

To track the changes of heterogeneity between stages, we plotted a heatmap of methylation heterogeneity using both tumour DHGs and adjacent normal DHGs (Fig. 4C). The heatmap shows there are clear changes of methylation heterogeneity from normal, adjacent normal to tumours, where most of the genes increased their heterogeneity towards tumours. A similar heatmap on DMGs was not able to reveal the differences accurately between the sample groups, as one normal sample is classified within the cancer group (Additional file 1: Fig. S5). We analyzed the enriched functions of the non-overlapping DHGs specific to either adjacent normal (Additional file 1: Fig. S6) or tumour (Additional file 1: Fig. S7) via ingenuity pathway analysis [38]. The enriched diseases and functions clearly indicated that DHGs identified by comparing adjacent normal samples against normal samples were involved in colorectal cancer-associated diseases; suggesting the changes of methylation heterogeneity at these genes are highly associated with the cancer progression, and the adjacent normal DHGs are predictive of CRC tumours. In summary, the DHG analysis complements conventional DMG approaches in the selection of regions associated with phenotypes of interest.

### Identifying specific methylation patterns associating with increased heterogeneity

In total we identified 162 genes overlapping significantly between the tumour DHGs and adjacent normal DHGs (test for overlapping; *p* < 0.00001) (Fig. 4B; Additional file 1: Fig. S8). These genes showed strong and persistent changes of heterogeneity in DNA methylation towards tumour formation. The change of methylation heterogeneity at these genes may be suggesting specific methylation patterns emerging with the changes of cell types due to cancer formation or cell differentiation for example.

As an example, we found *CPXM2* from the 162 overlapping DHGs. *CPXM2* is a protein-coding gene that has been reported to be associated with several human disorders such as developmental diseases [39], Alzheimer’s disease and schizophrenia [40], and to promote tumour aggressiveness when active [41]. As shown in the screenshot of methylation heterogeneity at *CPXM2* (Fig. 4D), an overlapping DHRs was constantly found at the promoter for comparisons between adjacent normal, and normal samples and between tumour and normal samples. The compositions of the methylation patterns at this particular DHR (Fig. 4E) revealed a specific methylation pattern labelled ‘1111’ in orange colour (fully methylated cytosines in a row) that seemed to be a “disease” pattern. It was not present in normal samples, but it started to appear in adjacent normal samples and became stabilized in tumour samples. Moreover, the proportions of reads showing this pattern increased in the presence of either an increased fully methylated ‘1111’ pattern or other partially methylated patterns that closely resembled ‘1111’ such as ‘0111’ or ‘1011’…etc.; instead of patterns resembling unmethylated ‘0000’, which was observed for most patterns in normal samples, the reads began to become similar to pattern ‘1111’. This verifies the ability of our model to detect changes in methylation patterns, which may serve as biomarkers for the early detection of disease.

## Discussion

In this study, we proposed an approach adopted from Chao et al*.*’s mathematical framework [26] for biodiversity to estimate methylation heterogeneity. Our model-based methods were subsequently implemented as MeH program to estimate genome-wide methylation heterogeneity from methyl-seq data. Our results demonstrated the ability of MeH to highlight different methylation patterns across multiple subpopulations of methylomes. Unlike the existing approaches that only detect distinct patterns, our methods based on Hill numbers are equipped with mathematical properties for achieving unbiased estimation and are enabled for analyzing pattern similarity between reads. Furthermore, MeH can be used as a tool for evaluating CG as well as non-CG methylation heterogeneity, and interrogating the changes in methylation patterns among prespecified cell populations during cancer development.

### Improvement from the existing estimators

Our analyses revealed that some of the existing methylation heterogeneity methods give scores of nonlinearity, and some do not accommodate similarities between methylation patterns; both lead to a less favourable outcome. In other words, when evaluating methylation heterogeneity using methods such as those based on ME or EP, changes tend to be overestimated when the number of distinct patterns is small and underestimated when the number of distinct patterns is large in comparisons between samples or genomic locations. There will be variations among the differences in scale, which may result in false findings (of significant changes) if we treat them equally. The ignorance of pattern similarity in other scores, such as the concordance-based methods, could in principle be modified to take it into account in the future development. In addition, as sequencing errors have been shown to introduce biases in heterogeneity scores [18], the behaviour of the scores including PWS, ME and MP were evaluated using simulated bisulfite sequencing data (see Additional file 1: Note S4). While all methods are affected by the sequencing errors, PWS show minor changes (3.7%) when introducing sequencing errors of 5%, with a lower variation among replicates. Considering the general sequencing error nowadays is less than 1–2%, PWS remains a good choice of the methods.

On the other hand, one caveat of the methods that implement window-based screening (such as model-based, ME and EP) is that only complete methylation patterns (i.e., fully aligned reads) are considered. This may lead to significant data loss. However, with the current NGS technology, the commonly used read length of 150–200 base pairs is sufficient to cover four CpG dinucleotides, and the cost of sequencing has significantly decreased in the past years. There are also imputation tools that can overcome the problem of low coverage by adding simulated reads mimicking the neighbouring methylation patterns, such as BSImp [42, 43], to maximise the use of data with limited sequencing depth.

We developed three variants based on a framework that considers different attributes for quantifying methylation heterogeneity using methylation patterns and demonstrated each variant’s strengths and weaknesses together with the evaluation of existing methods using both toy examples and single-cell methylomes. Whilst every method has its own weaknesses and some are highly correlated, none are replaceable. A thorough comparison was conducted by [18] and indicated the scenarios when each score should be used. In particular, we showed that our models ameliorated the nonlinearity problem and were thus advantageous for systematic genome-wide comparisons across samples and for identifying DHRs for further analyses.

### Validation against pooled single-cell methylomes

Following our analysis using single-cell methylomes, we also demonstrated the potential of the method for estimating cell-type compositions based on bulk versus merged methylomes, as the effect seems to be addictive. We expected that adding additional single-cell methylomes would result in increased methylation heterogeneity, which could be approximated by taking the difference between genome-wide methylation heterogeneity estimated using 8 and 6 single-cell methylomes (Fig. 2E). Then, given the genome-wide methylation heterogeneity of a specific methylome, we could potentially estimate cellular compositions. Such analysis for one type of cell here but could eventually elaborate the strategy to incorporate different types of cells (Fig. 2D), which might be beneficial for studies such as those distinguishing the compositions of cellular populations to improve cellular differentiation accuracy in the context of disease or development.

### Importance of the consideration of similarities between methylation patterns

The compositions of methylation patterns presented in Fig. 4E demonstrate the potential of using DHR for identifying the possible presence of a specific methylation pattern. Such methylation patterns at specific DHR locations could be considered biomarkers for particular phenotypes. The ‘1111’ pattern of fully methylated cytosines appeared in a very low proportion of adjacent normal and tumour samples and only appeared after a large proportion of the methylation patterns were changed from those commonly seen in the normal. This has two implications. First, either the change in methylation patterns is a gradual process, or the appearance of methylation patterns relies on the presence of an intermediate pattern. In either case, the methylation heterogeneity is likely to be more sensitive than the methylation levels. This further indicates the importance of considering similarities between methylation patterns in quantifying methylation heterogeneity, as not all patterns are equally distinctive.

### Direct association between phenotypes and methylation heterogeneity

It is often difficult to identify the association between methylation and gene expression, let alone the association between methylation and phenotypes based on gene expression; however, methylation heterogeneity is thought to be directly associated with phenotypes, so the assumption was made, and the data were analyzed. Again, this method is independent of methylation levels and was verified to be so. Although we were not investigating the mechanism of epigenetic regulation here, the methylation profiles in *Arabidopsis* illustrated the negative correlation between methylation heterogeneity and the expression of gene and TE that may actually open up a new direction for methylation analysis jointly with the transcriptome. Lastly, the tracking of patterns in the colorectal cancer example does show that methylation heterogeneity can be a more effective indicator than methylation levels when studying disease progression.

## Conclusions

**Fig. 5:**
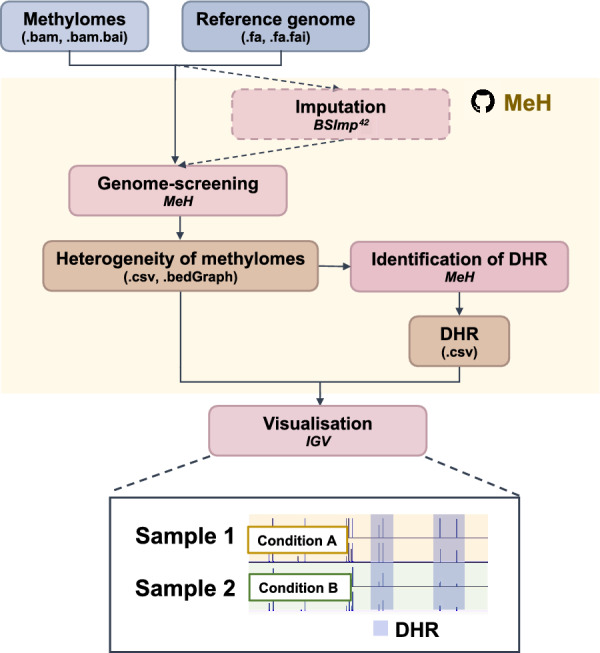
Overview of MeH workflow. The functions by MeH are shown in the yellow area. Each box represents a component corresponding to a series of tasks. Data processing steps are indicated in red, the tools employed in the step are indicated in italics, and the dotted box means that the step is optional. The file types are indicated in brackets for input data (blue boxes) and output data (orange boxes)

Ultimately, MeH (workflow illustrated in Fig. 5) can be employed to profile genome-wide methylation heterogeneity using proposed model-based methods. This method provides users with the freedom to specify window size in terms of the number of cytosine sites and methylation contexts, including all CG, CHG, and CHH contexts, for the evaluation of methylation heterogeneity, and is the first of its kind. We also embedded a methylome imputation method that was developed recently [42] to maximize coverage for the evaluation of methylation heterogeneity with limited bias, as demonstrated in Additional file 1: Fig. S9. It can impute the methylation statuses with over 85% accuracy and result in only ~ 3% of bias when estimating methylation level. Although many studies on cellular heterogeneity have focused on mammalian data, important studies of topics such as methylation regulation, which involves different DNA methyltransferases, can only be performed on plants, in which methylation is common in other contexts, such as CHG and CHH [44]. Finally, we provided an example of the application of a mathematical model developed for biodiversity in the estimation of methylation heterogeneity.

## Methods

### Model-based methods for measuring methylation heterogeneity

The estimation of DNA methylation heterogeneity is mostly based on the observation of methylation patterns in terms of read patterns (Fig. 1B). The similarities between patterns, such as the distances between methylation patterns and branch lengths within phylogenetic trees constructed using distances between read patterns, may also be instrumental for assessing the process of epigenomic changes. We adopted three models from Chao’s unified framework [26]: the *abundance-based heterogeneity* model (AB), *pairwise-similarity-based heterogeneity* model (PWS) and *phylogenetic-tree-based heterogeneity* model (PHY), for estimating DNA methylation heterogeneity based on Chao et al*.*’s three variant diversity models, species diversity, phylogenetic diversity and the distance-based functional diversity.

The AB method uses the relative abundances of read patterns to provide a measure of heterogeneity. While considering the changes in methylation status within a certain genomic location in a cell is a gradual process, the similarity between two methylation patterns can be assessed based on two different subtypes of cells with a certain degree of similarity or similar stages of differentiation. We incorporate pattern similarity for assessing methylation heterogeneity in both the PWS and PHY methods, where the former considers pairwise distances independently, and the latter considers the relationships between all patterns at once via the construction of a phylogenetic tree (Additional file 1: Fig. S10. Following Chao et al*.*’s base model in Eq. (1) with $q=2$ for giving a robust estimate in different situations, we introduce our AB, PWS and PHY methods as follows.

### Abundance-based heterogeneity

If we consider $C$ as a set (collection) of distinct DNA methylation patterns; $u$ as any pattern in set $C$; ${a}_{u}$ as the absolute abundance of $u$ (*i.e.*, the number of reads with the same pattern, $u$), providing values for distinct patterns; and $\overline{V }$ as a normalising factor, we define DNA methylation heterogeneity as follows under the AB model:

2 $$AB={\left(\sum_{i=1}^{R}{p}_{i}^{2}\right)}^{-1}$$

where $R$ is the number of distinct methylation patterns, $pi$ is the relative abundance for pattern $i$ (*i.e.*, the number of reads equal to pattern $i$ over the total number of reads). AB heterogeneity considers individual methylation patterns as attribute, which uses weights of distinct methylation patterns and here we used 1 for all methylation patterns. AB provided the effective number of patterns as a weighted average of the abundances of each pattern.

### Pairwise-similarity-based heterogeneity

To consider pattern similarity, PWS uses pairs of methylation patterns as attribute, instead of individual methylation patterns used in AB. In PWS, the pairwise distances between any two methylation patterns are calculated using the Hamming index and the weighted degree kernel [45] as introduced in Additional file 1: Note S2. These distances are incorporated into the general model as attribute values. Let $S$ be the total number of pairs of methylation patterns, ${p}_{ij}$ be the attribute abundance of pattern pairs between pattern $i$ and pattern $j$, ${p}_{i}$ is the relative abundance for pattern $i,$ and $dij$ be the distance between patterns $i$ and$j$. PWS may be measured as follows:

3 $$PWS={\left(\sum_{i, j=1}^{S}{{d}_{ij}\times p}_{ij}^{2}\right)}^{- \frac{1}{2}}$$

where

4 $${p}_{ij}=\frac{{p}_{i}{p}_{j}}{\sum_{i, j=1}^{S}{d}_{ij}{{p}_{i}p}_{j}}=\frac{{p}_{i}{p}_{j}}{Q}$$

This estimate can be interpreted as the effective sum of pairwise distances between methylation patterns. The method differs from the unifying framework in that we took the square root when estimating diversity. This is because the doubling property (Additional file 1: Note S1) of this variant results in quadrupled diversity after the combination of two groups with the same diversity and $Q$ (a multiple of the expected pairwise distances between methylation patterns, as shown in Eq. (4). The reason for this is that the sum of pairwise distances between two groups of methylation patterns equals the sum of (1) the sum of pairwise distances within the groups and (2) the sum of pairwise distances between the groups, which is quadrupled (assuming the sums are all the same and have the same $Q$).

### Phylogenetic-tree-based heterogeneity

A phylogenetic tree was constructed in which each node represented one methylation pattern, allowing us to estimate overall heterogeneity in terms of branch length and the corresponding branch abundances. Here, the phylogenetic tree is constructed using $dij$, representing the pairwise distances between distinct patterns calculated as shown in the PWS method. If there are B branch segments in the tree, $Li$ is the length of branch $i$ and $pi$ is the branch abundance associated with branch $i$, the PHY can be calculated as follows:

5 $$PHY={\left(\sum_{i=1}^{B}{{L}_{i}a}_{i}^{2}\right)}^{-1}$$

where

6 $${a}_{i}=\frac{{p}_{i}}{\sum_{j=1}^{B}{L}_{j}{p}_{j}} .$$

The attribute values of PHY heterogeneity are the branch lengths within a phylogenetic tree that is constructed using pairwise distances between patterns. To illustrate our method, we considered five DNA methylation patterns as 5 nodes in the phylogenetic tree (Additional file 1: Fig. S10). The tree could be constructed given ${C}_{2}^{5}$ = 10 pairwise distances between the methylation patterns. Five branches are connected to the five nodes. With PHY, the attribute abundances were associated with these branches that were equal to the abundances of the nodes to which they were connected. For other branches, the attribute abundance was calculated as the sum of the abundances of the subbranches. For example, for the branch with length $L6$, the subbranches were node 2 and node 3, which were associated with abundances of $p2$ (raw abundance of pattern 2 at the loci) and $p3$. Then, the abundance associated with L_6_ was $p6 = p2+p3$. Therefore, the set of branch abundances was expanded from $\left\{p1,p2,\dots ,pR\right\}$ to $\left\{p1,\dots ,pR,pR+1,\dots ,pB\right\}$, where $R$ is the number of distinct patterns (nodes). In Additional file 1: Fig. S10, $R$ is 5 and $B$ is 8.

### Alignment and processing of methylome data

High-quality cleaned Illumina paired-end reads were aligned to the reference genomes (TAIR10, hg19 and mm10 for *Arabidopsis*, cancer and single-cell data, respectively) using BS-Seeker2 [46] and BSBolt [47]. Only uniquely mapped reads were included in the analyses. DNA methylation levels were calculated as $\left(\frac{\#C}{\#C+\#T}\right)$, with coverage by at least four reads in all cases for accurate estimation.

Our criteria for calling regions of differential methylation (DMRs) were as follows: (1) the difference between the mean DNA methylation levels of the samples was greater than 15% and (2) the Student’s *t*-test *p*-value was less than 5%. For the identification of DMRs throughout the genome, regions containing at least five cytosines within 400-bp tiles were first identified and were further defined according to the two aforementioned criteria. Genes with DMR located in the genebody were identified as differentially methylated genes (DMGs). Methylation heterogeneity was profiled using our own program, MeH, and was evaluated using PWS heterogeneity. Removing duplicated reads is recommended to avoid potential bias caused by PCR amplification.

Methylation heterogeneity was evaluated using sliding windows of 4 cytosines given that enough reads were included within the window (depth ≥ 4 reads), and the results were then merged into 400-bp tiles. Differentially heterogeneous regions (DHRs) were called based on the following criteria: (1) the difference between the mean methylation heterogeneity of samples was greater than 1.41 which corresponds to the expected increase in estimated heterogeneity when adding a new methylation pattern; and (2) Student’s *t*-test *p*-value < 5%. See Additional file 1: Fig. S4 for the Venn of DHRs identified from the CRC samples. Genes with DHRs located in the genebody were identified as differentially heterogeneous genes (DHGs).

### Ingenuity pathway analysis

DHGs and DMGs between adjacent normal and normal samples and between tumour and adjacent normal samples were screened using common regions of 400 bp with data on both methylation levels and methylation heterogeneity. There were 8074 regions located within genebodies. A total of 660 and 455 DHGs and 10 and 4 DMGs were identified at adjacent normal and at tumour, respectively (see Fig. 4B). Disease and functional analyses were performed using the threshold of an FDR < 0.05.

### Supplementary Information

**Additional file 1: Figure. S1.** Schematic illustration of linear and non-linear scores in estimating methylation heterogeneity. When the score increases by the same value K, the corresponding changes of heterogeneity are not equal (h1≠ h2), indicating nonlinearity of the score. **Figure. S2.** Genome-wide methylation heterogeneity and methylation level of *A. thaliana *at CG, CHG and CHH. **Figure. S3.** The methylation heterogeneity profile of *A. thaliana *at CG and non-CG sites. **A** Enrichment plots of high (top 10%) and low (bottom 10%) heterogeneity regions across different genomic features. **B** Metagene plot of methylation heterogeneity profile for highly and lowly expressed genes (top and bottom 25%), as well as their 4 kb upstream TSS and 4kb downstream of TES. **C** Meta plots of methylation heterogeneity for highly and lowly (top and bottom 25%) expressed TEs and their neighbouring regions. **Figure. S4.** The Venn diagram of the regions found as DMRs and DHRs. **Figure. S5.** The results of CRC DMGs analyses. The heatmap of methylation level of DMGs in normal, adjacent normal and tumour samples. **Figure. S6.** Disease and functional analysis for adjacent normal DHGs. The red line represents the adjusted *p*-value < 0.05 and the red-shaded texts are those diseases related to colon cancer. **Figure. S7.** Disease and functional analysis for tumour DHGs. The red line represents the adjusted *p*-value < 0.05 and the red-shaded texts are those diseases related to colon cancer. **Figure. S8.** The results of CRC DHGs analyses using ME. **A** The Venn diagram of DHGs found by ME. **B** The results comparisons of overlapping DHGs identified by ME and PWS. **C** The heatmap of ME methylation heterogeneity of DHGs in normal, adjacent normal and tumour samples. **Figure. S9.** Effect of the imputation of methylation heterogeneity using PWS heterogeneity. Each dot represents the mean methylation heterogeneity of 2 replicates. The black lines represent the median values of the data. **Figure. S10.** Example of methylation patterns and the parameters within the PHY heterogeneity. Phylogenetic tree was constructed using 5 distinct patterns as an illustration of how the parameters are obtained in the formula when estimating PHY heterogeneity; ‘p’ represents the abundance used in the formula, and ‘L’ represents the branch length in the tree; these values are also used in the formula. **Table S1.** The runtime of different methylation heterogeneity methods. **Note S1.** Mathematical properties of the mathematical framework. **Note S2.** Distance between methylation patterns. **Note S3.** Testing the linearity of MeH with single-cell methylomes. **Note S4.** Evaluation of scores using simulated methylomes with sequencing errors.

## Data Availability

All sequencing data used in the study were downloaded from the NCBI Gene Expression Omnibus under accession numbers GSE197898 and GSE39901 for *Arabidopsis* [34, 48], GSE121436 and GSE56879 for single-cell methylomes [10, 49] and GSE95656 for cancer data [35]. The annotations of *Arabidopsis* genomic features were downloaded from The Arabidopsis Information Resource (TAIR) [50], while the locations of *Arabidopsis* enhancers were the DNase I hypersensitive [4] sites identified by Zhang [51]. MeH has been implemented in R and Python and released at https://github.com/PaoyangLab/MeH. Users are able to input there.bam and.bai files of methylome for genome screening and receive the.csv and.bedGraph files as genome-wide CG/CHG/CHH methylation heterogeneity output for visualization. We recommend removing duplicated reads in the.bam file by Samtools before running MeH [52]. Three proposed models are all available in MeH for calculating genome-wide heterogeneity. DHRs can be detected when input multiple samples are after genome screening, returning the.csv file as a list of DHRs and DHGs. We provide toy examples and a step-by-step tutorial for users to get started with MeH on GitHub. For reference, calculating the methylation heterogeneity for the *Arabidopsis* genome using PWS took approximately 131 min using 44 CPU cores and 100 GB of memory resources, see Additional file 1: Table S1. **Software:** Project name: MeH; Project home page: https://github.com/PaoyangLab/MeH; Operating system(s): Linux, MacOSX; Programming language: R and Python; License: MIT License; Any restrictions to use by non-academics: license needed.

## Abbreviations

AB: Abundance-based heterogeneity
BS-seq: Bisulfite sequencing
CRC: Colorectal cancer
DHG: Differentially heterogeneous genes
DHR: Differentially heterogeneous region
DMG: Differentially methylated gene
DMR: Differential methylation region
EM-seq: Enzymatic methyl sequencing
ESC: Embryonic stem cell
EWAS: Epigenome-wide association studies
FDRP: Fraction of discordant read pairs
IGR: Intergenic regions
ME: Methylation entropy
MHL: Methylation haplotype load
NGS: Next-generation sequencing
PHY: Phylogenetic-tree-based heterogeneity
PDR: Proportion of discordant reads
EP: Epipolymorphism
PWS: Pairwise-similarity-based heterogeneity
qFDRP: Quantitative FDRP
TE: Transposable elements
