# Supplementary material for: Estimating genome-wide DNA methylation heterogeneity with methylation patterns
Source: Epigenetics Chromatin. 2023 Nov 9;16:44. doi: 10.1186/s13072-023-00521-7 (PMC10634068; doi:10.1186/s13072-023-00521-7)
Supplement: Supplementary file 1 — Additional file 1: Figure. S1. Schematic illustration of linear and non-linear scores in estimating methylation heterogeneity. When the score increases by the same value K, the corresponding changes of heterogeneity are not equal (h1≠ h2), indicating nonlinearity of the score. Figure. S2. Genome-wide methylation heterogeneity and methylation level of A. thaliana at CG, CHG and CHH. Figure. S3. The methylation heterogeneity profile of A. thaliana at CG and non-CG sites. A Enrichment plots of high (top 10%) and low (bottom 10%) heterogeneity regions across different genomic features. B Metagene plot of methylation heterogeneity profile for highly and lowly expressed genes (top and bottom 25%), as well as their 4 kb upstream TSS and 4kb downstream of TES. C Meta plots of methylation heterogeneity for highly and lowly (top and bottom 25%) expressed TEs and their neighbouring regions. Figure. S4. The Venn diagram of the regions found as DMRs and DHRs. Figure. S5. The results of CRC DMGs analyses. The heatmap of methylation level of DMGs in normal, adjacent normal and tumour samples. Figure. S6. Disease and functional analysis for adjacent normal DHGs. The red line represents the adjusted p-value < 0.05 and the red-shaded texts are those diseases related to colon cancer. Figure. S7. Disease and functional analysis for tumour DHGs. The red line represents the adjusted p-value < 0.05 and the red-shaded texts are those diseases related to colon cancer. Figure. S8. The results of CRC DHGs analyses using ME. A The Venn diagram of DHGs found by ME. B The results comparisons of overlapping DHGs identified by ME and PWS. C The heatmap of ME methylation heterogeneity of DHGs in normal, adjacent normal and tumour samples. Figure. S9. Effect of the imputation of methylation heterogeneity using PWS heterogeneity. Each dot represents the mean methylation heterogeneity of 2 replicates. The black lines represent the median values of the data. Figure. S10. Example of methylation patterns [file 13072_2023_521_MOESM1_ESM.pdf]

# Supplementary Figures

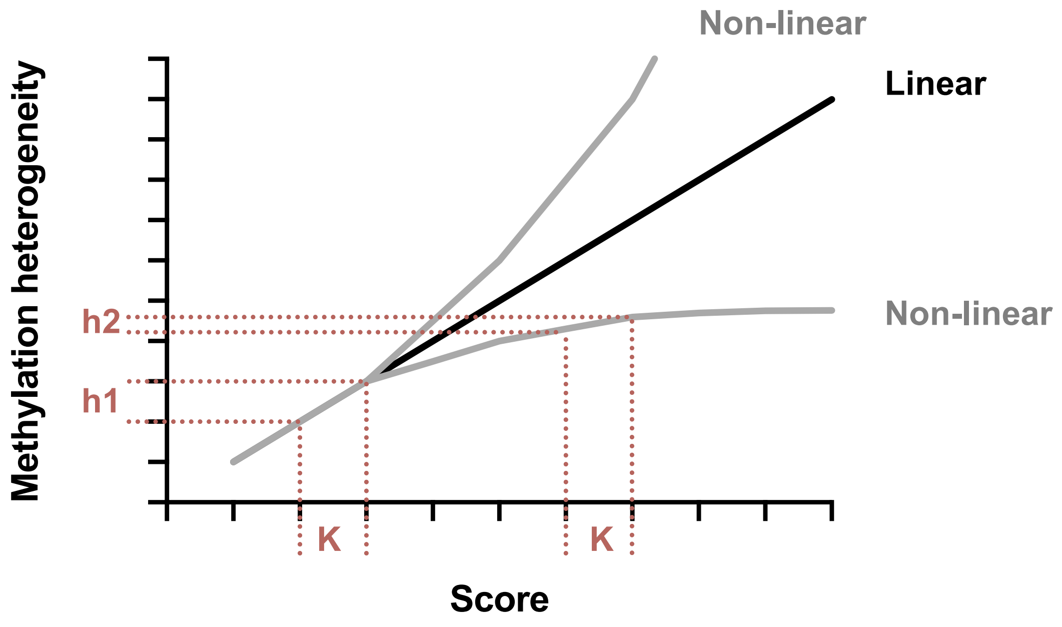


**Fig. S1.** Schematic illustration of linear and non-linear scores in estimating methylation heterogeneity. When the score increases by the same value K, the corresponding changes of heterogeneity are not equal (h1≠ h2), indicating nonlinearity of the score.

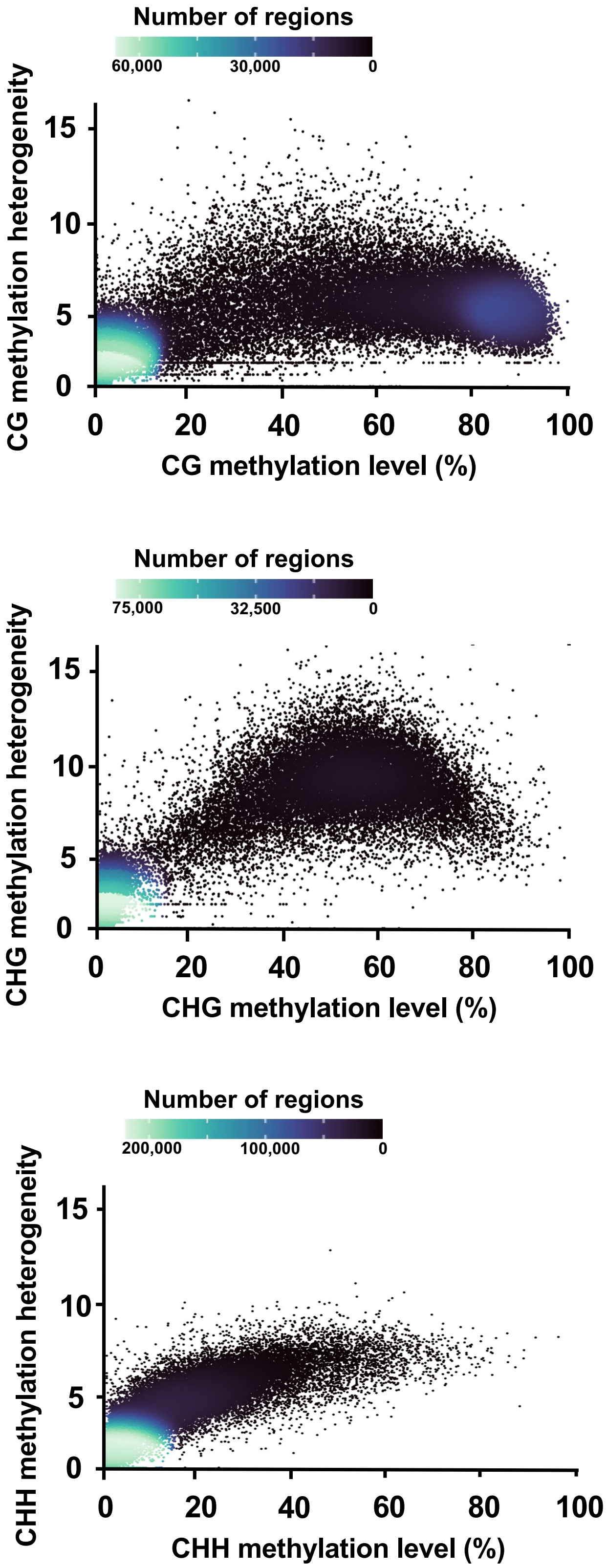


**Fig. S2.** Genome-wide methylation heterogeneity and methylation level of *A. thaliana* at CG, CHG and CHH.

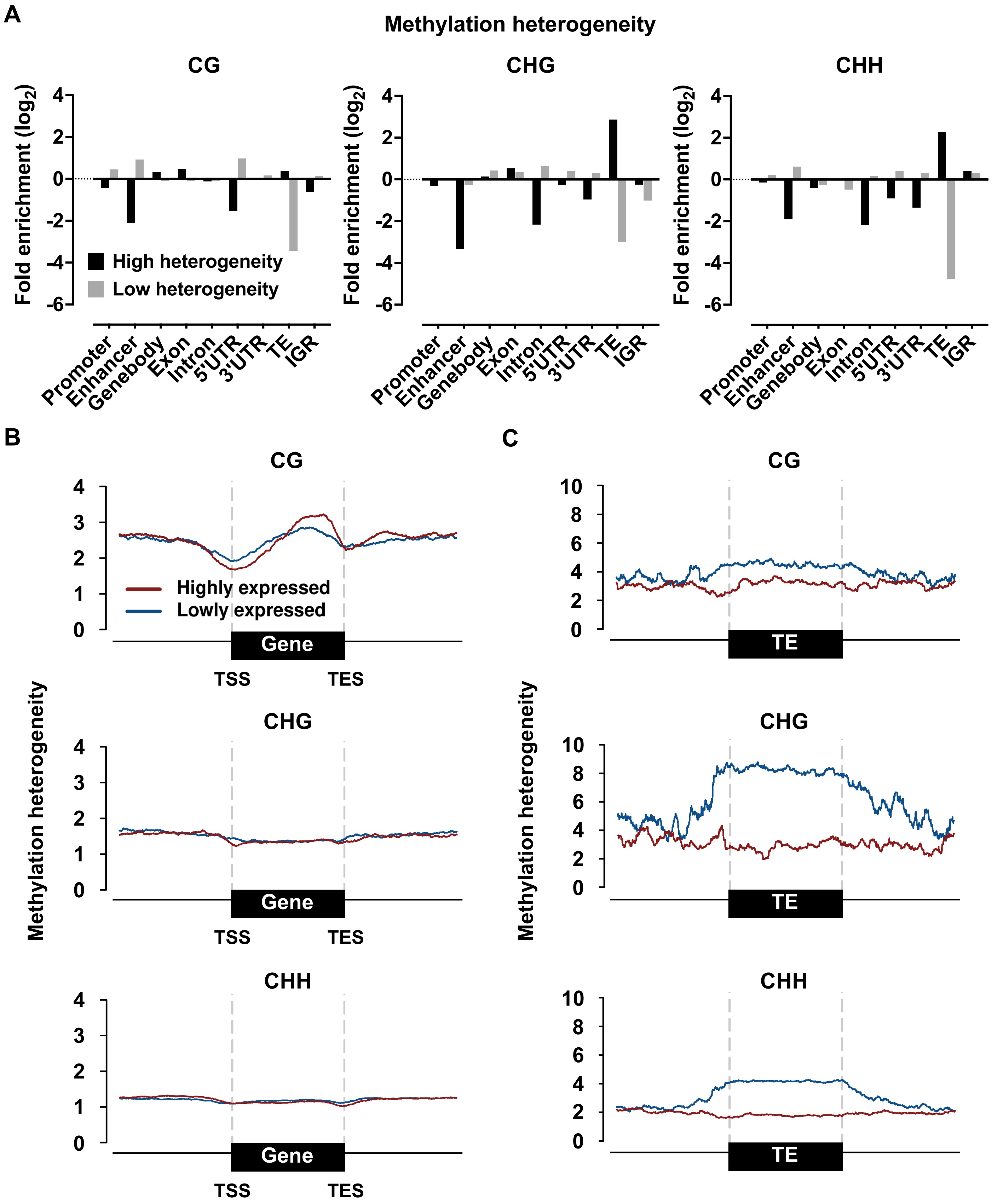


**Fig. S3.** The methylation heterogeneity profile of *A. thaliana* at CG and non-CG sites. (A) Enrichment plots of high (top 10%) and low (bottom 10%) heterogeneity regions across different genomic features. (B) Metagene plot of methylation heterogeneity profile between highly and lowly expressed genes (top and bottom 25%). (C) Meta plots of methylation heterogeneity between highly and lowly (top and bottom 25%) expressed TEs and their neighbouring regions.

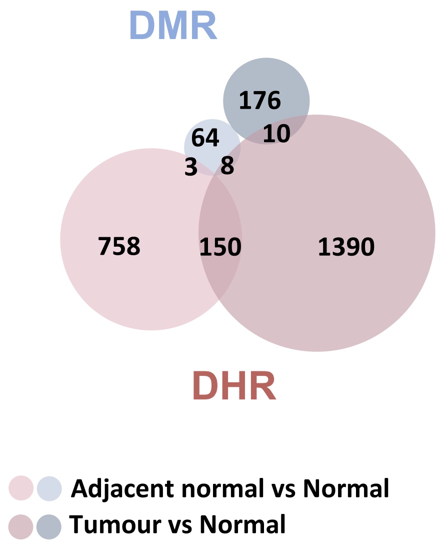


**Fig. S4.** The Venn diagram of the regions found as DMRs and DHRs.

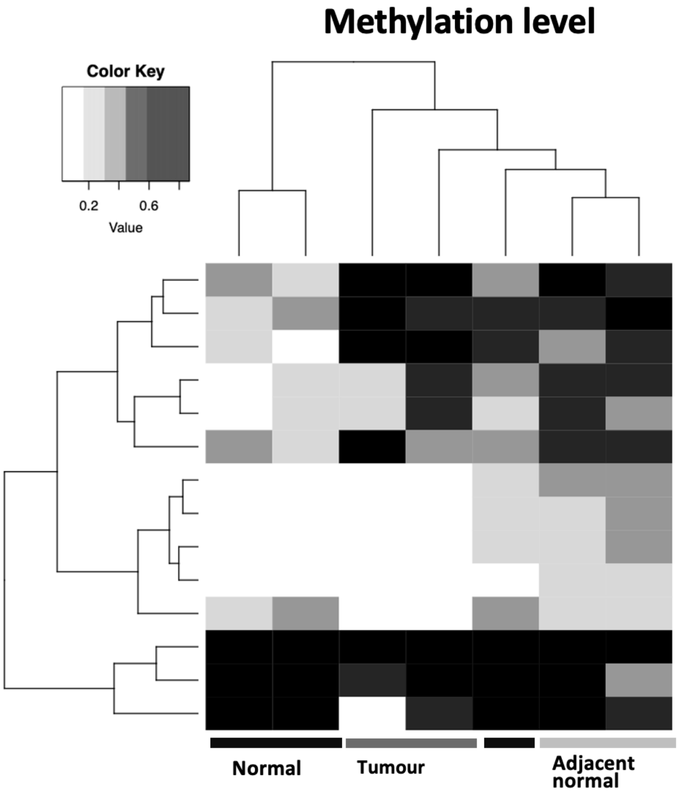


**Fig. S5.** The results of CRC DMGs analyses. The heatmap of methylation level of DMGs in normal, adjacent normal and tumour samples.

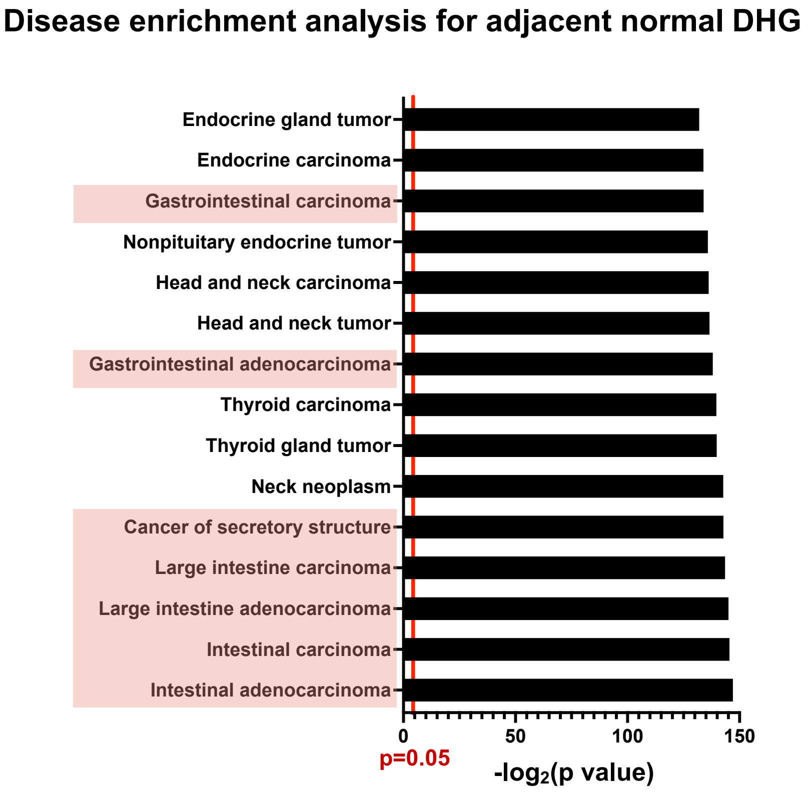


**Fig. S6.** Disease and functional analysis for adjacent normal DHGs. The red line represents the adjusted p value < 0.05 and the red-shaded texts are those diseases related to colon cancer.

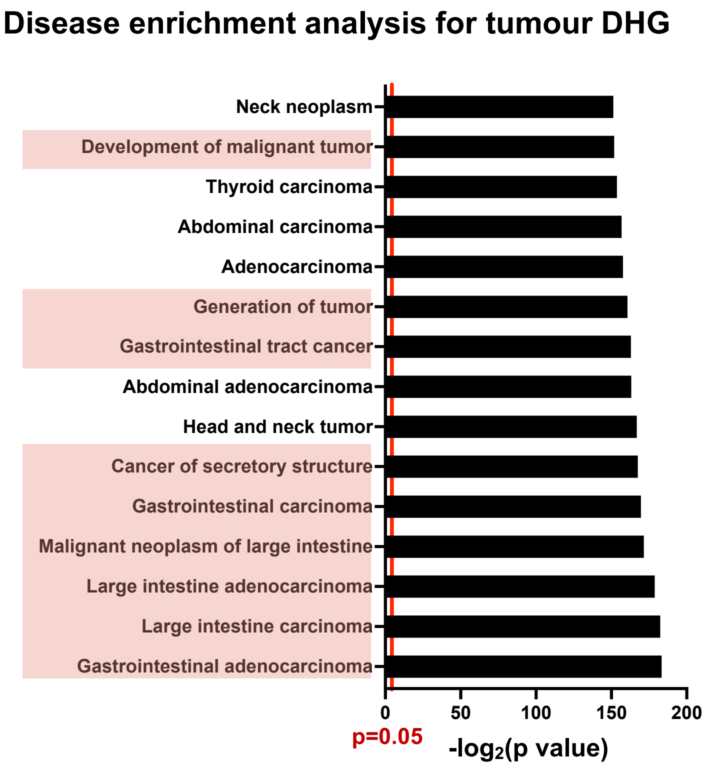


**Fig. S7.** Disease and functional analysis for tumour DHGs. The red line represents the adjusted p value < 0.05 and the red-shaded texts are those diseases related to colon cancer.

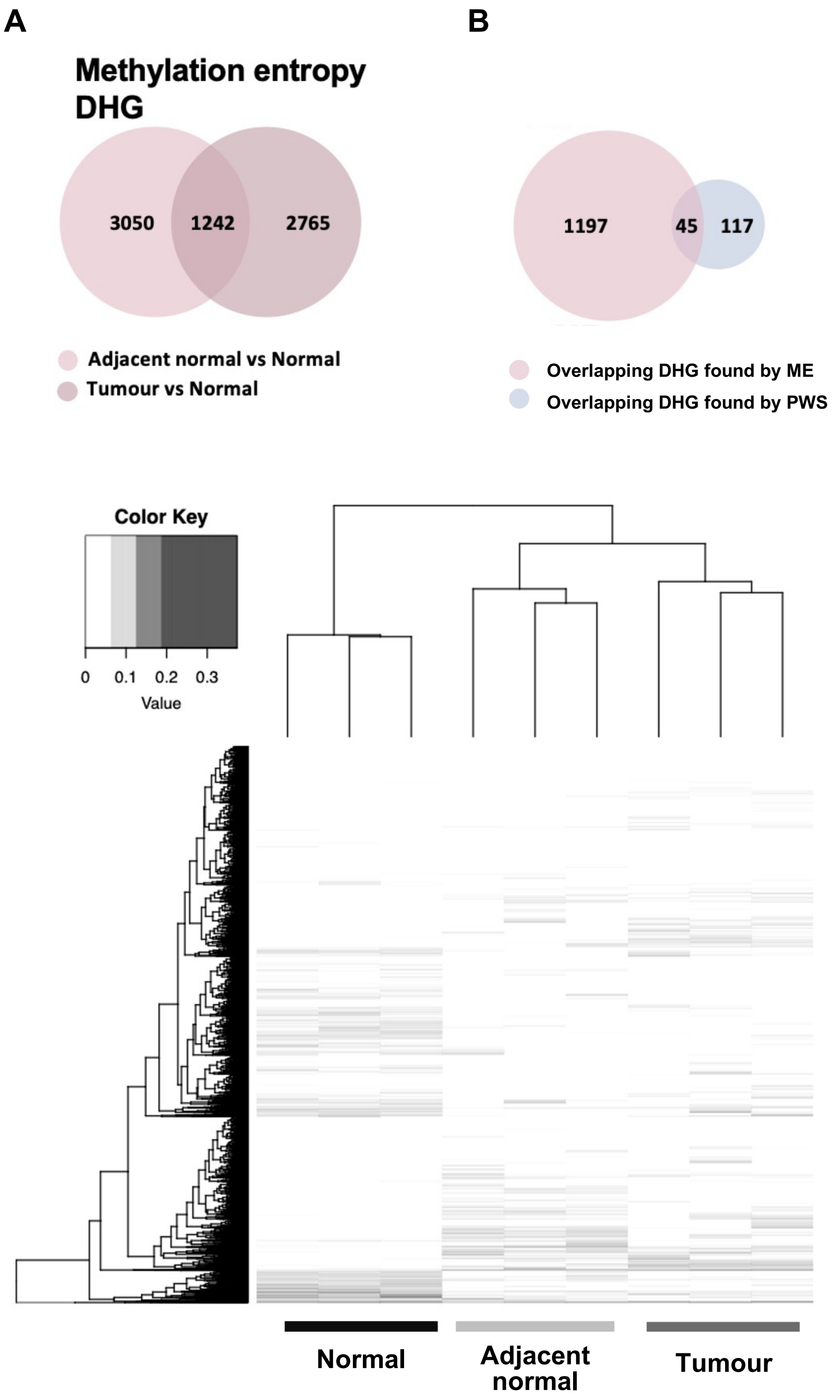


**Fig. S8.** The results of CRC DHGs analyses using ME. (A) The Venn diagram of DHGs found by ME. (B) The results comparisons of overlapping DHGs identified by ME and PWS. (C) The heatmap of ME methylation heterogeneity of DHGs in normal, adjacent normal and tumour samples.

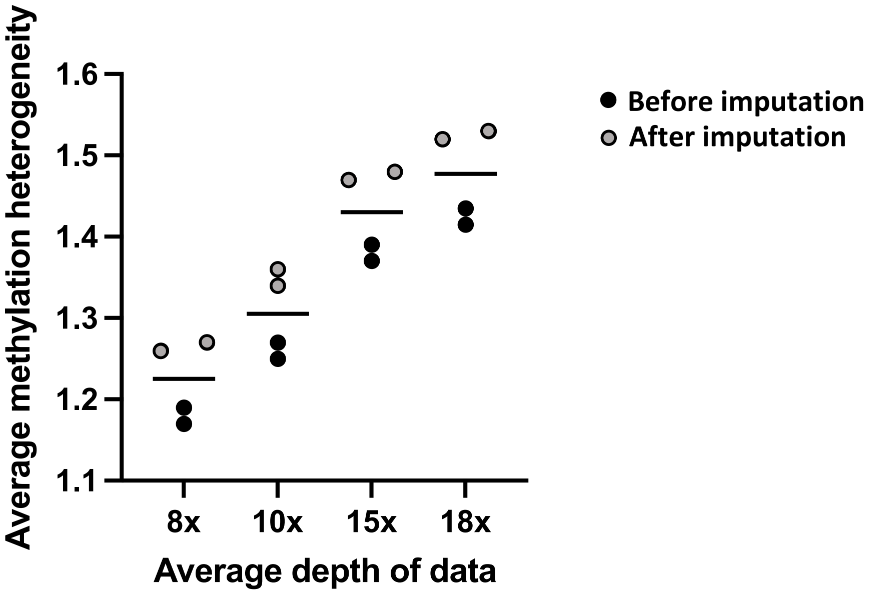


**Fig. S9.** Effect of the imputation of methylation heterogeneity by using PWS heterogeneity. Each dot represents the mean methylation heterogeneity of 2 replicates. The black lines represent the median values of the data.

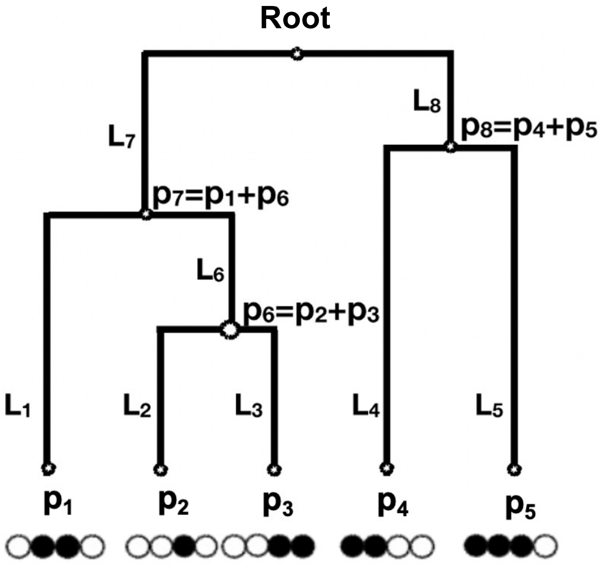


**Fig. S10.** Example of methylation patterns and the parameters within the PHY heterogeneity. Phylogenetic tree was constructed using 5 distinct patterns as an illustration of how the parameters are obtained in the formula when estimating PHY heterogeneity; ‘p’ represents the abundance used in the formula, and ‘L’ represents the branch length in the tree; these values are also used in the formula.

# Supplemental Table

**Table S1.** The runtime of different methylation heterogeneity methods.

| Methods | Tool | Runtime (minute) [2] |
|---|---|---|
| qFDRP | R package "WSH[1]", compute.score() function with score argument "qfdrp" | 232.3 |
| PDR | R package "WSH", compute.score() function with score argument "pdr" | 3.4 |
| MHL | R package "WSH", compute.score() function with score argument "mhl" | 9304.3 * |
| ME | R package "WSH", compute.score() function with score argument "entropy" | 1.1 |
| PWS | MeH | 131 |

1. Tested by data from an *Arabidopsis* methylome with a genome size of 125Mbp (1.4M reads from GSE39901), and the same server of 44 CPU cores and 100 GB of memory.

# Supplemental Notes

## Note S1 Mathematical properties of the mathematical framework

1. Scale invariant
   The phylogenetic Hill numbers are invariant to the units used to measure branch lengths.

2. Doubling (replication principle)
   Hill proved a weak version of the replication principle [1]: If N equally large and completely distinct assemblages (*i.e.*, no species in common) have identical relative abundance distributions, then the Hill number of the pooled assemblage will be N times the diversity of an individual assemblage. Chiu et al. [2] proved a strong version of the doubling property: Given N equally diverse, equally large assemblages with no species in common (unlike the weak version, relative abundance distributions may be different), then the diversity of the pooled assemblage will be N times the diversity of a single assemblage. Thus, Hill numbers are linear under this kind of pooling of assemblages.

3. Weak monotonicity
   The measure does not decrease regardless of the distance matrices if a rarest new species is added to an assemblage [2, 3].

## Note S2 Distance between methylation patterns

To incorporate similarity or distance when evaluating methylation heterogeneity, we consider two existing methods in information theory. They can be easily applied to evaluate distances between any two methylation patterns.

Hamming distance

The Hamming distance [4] between two methylation patterns of equal length is calculated as the number of positions at which the methylation status differs. This method computes pairwise mismatches between two methylation patterns of equal length.

Weighted degree kernel

The weighted degree kernel (WDK)[5] is a measure of similarity between sequences or methylation patterns that takes positional information into account. Let $u_{kl}(s)$ denote the subsequence or subpatterns of length k starting at the $l$−th position of pattern s; the indicator function $I(u_{kl}(x_i) = u_{kl}(x_j))$ is a measure of whether subpatterns $u_{kl}(x_i)$ and $u_{kl}(x_j)$ equate. Using WDK, the distance between DNA methylation patterns can be calculated as follows:

$$d_{x_i,x_j} = \sum_{i<j} (V - k(x_i, x_j))$$

(Equation 7)

where

$$k(x_i, x_j) = \sum_{k=1}^{L} (L-k) \sum_{l=1}^{L-k+1} I(u_{kl}(x_i) = u_{kl}(x_j))$$

(Equation 8)

and

$$V = \sum_{k=1}^{L} (L-k) \sum_{l=1}^{L-k+1} 1 = \sum_{k=1}^{L} (L-k)\,(L-k+1)\,.$$

(Equation 9)

equals the maximum possible $k(x_i, x_j)$ (if $x_i = x_j$). Unlike Hamming distance, WDK considers the similarities of methylation patterns by comparing their subpatterns when measuring distances.

## Note S3 Testing the linearity of MeH with single-cell methylomes

We wanted to show the relationships between an increase in methylation heterogeneity and an increase in single cells in a methylome as a way of measuring the linearity of a score.

To do so, we first merged different numbers (6, 8, 10, 12, 14, 16, and 18) of single-cell methylomes by combining their methyl reads. These merged methylomes are referred to as methylomes A, B, C, D, E, F, and G. Every merged methylome contained two more single-cell methylomes than the previous methylome (*i.e.*, methylome B contained all methyl reads in methylome A). The resulting 7 merged methylomes were then aligned to the reference genome and screened for methylation heterogeneity for qualifying windows. For each merged methylome, we obtained a list of methylation heterogeneity results for windows with enough reads for each score. Common windows with methylation heterogeneity across all 7 merged genomes are noted. The methylation heterogeneity within these windows was summed within the same merged methylomes. The genome-wide methylation heterogeneity ratio was then calculated as the summed methylation heterogeneity divided by the summed methylation heterogeneity of the smallest merged methylome (methylome A), resulting in 7 values for each score, which were then plotted.

## Note S4 Evaluation of scores using simulated methylomes with sequencing errors

As the sequencing errors may result in perturbations of patterns when estimating heterogeneity. Simulated methylomes of 3 replicates with 0%, 5% and 10% error rate were generated. The changes in heterogeneity scores between those simulated methylomes with errors and those with no error were calculated. As shown in the figure below, we plotted the % change of the heterogeneity scores by comparing the methylomes with sequencing errors to ones with 0% error. These three methods PWS, ME, and EP are evaluated together as they shared similar input data format yet with different design principles.

While all methods are affected by the sequencing errors, the scores of ME change the most when there are 10% errors. EP showed a greater variation between replicates with 5% error. PWS show minor changes (3.7%) when introducing sequencing errors of 5%, with a lower variation among replicates. Considering the general sequencing error nowadays is less than 1-2%, PWS remains a good choice of the methods.

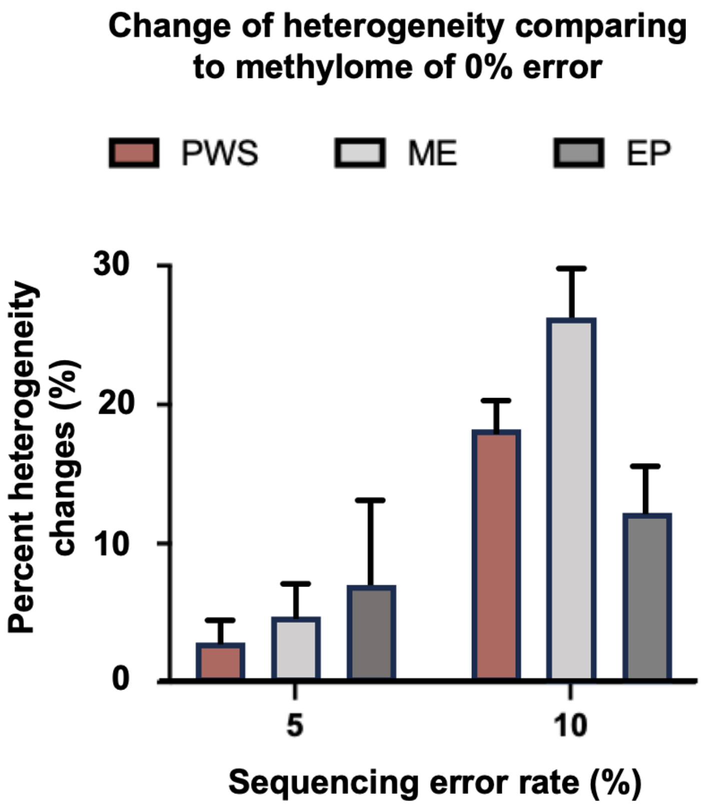

Note: The sequencing error data were generated using the pipeline from Scherer et al., 2020 [4] (https://github.com/ MPIIComputationalEpigenetics/WSHScripts). The data was simulated by Sherman v0.1.9 using human reference genome hg38. The simulated methylomes have depth of 24x.

## References


1. Hill MO. Diversity and evenness: a unifying notation and its consequences. Ecology. 1973;54(2):427-32.
2. Chiu C-H, Chao A. Distance-based functional diversity measures and their decomposition: a framework based on Hill numbers. PloS one. 2014;9(7):e100014.
3. Weikard HP, Punt M, Wesseler J. Diversity measurement combining relative abundances and taxonomic distinctiveness of species. Diversity and Distributions. 2006;12(2):215-7.
4. Scherer M, Nebel A, Franke A, Walter J, Lengauer T, Bock C, et al. Quantitative comparison of within-sample heterogeneity scores for DNA methylation data. Nucleic acids research. 2020;48(8):e46-e.
5. Sonnenburg S, Rätsch G, Schäfer C, Schölkopf B. Large scale multiple kernel learning. The Journal of Machine Learning Research. 2006;7:1531-65.
